# Wildlife population trends in protected areas predicted by national socio-economic metrics and body size

**DOI:** 10.1038/ncomms12747

**Published:** 2016-09-01

**Authors:** Megan D. Barnes, Ian D. Craigie, Luke B. Harrison, Jonas Geldmann, Ben Collen, Sarah Whitmee, Andrew Balmford, Neil D. Burgess, Thomas Brooks, Marc Hockings, Stephen Woodley

**Affiliations:** 1 School of Geography Planning and Environmental Management, the University of Queensland, St Lucia, Queensland 4067, Australia; 2 Australian Research Council Centre of Excellence for Environmental Decisions, the University of Queensland, St Lucia, Queensland 4072, Australia; 3 Australian Research Council Centre of Excellence for Coral Reef Studies, James Cook University, Townsville, Queensland 4811, Australia; 4 Redpath Museum, McGill University, 859 Sherbrooke Street West, Montreal, Quebec H3A 0C4, Canada; 5 Center for Macroecology, Evolution and Climate, Natural History Museum of Denmark, University of Copenhagen, Universitetsparken 15, Copenhagen E 2100, Denmark; 6 Conservation Science Group, Department of Zoology, University of Cambridge, Cambridge CB2 3EJ, UK; 7 Centre for Biodiversity and Environment Research, University College London, Gower Street, London WC1E 6BT, UK; 8 Indicators and Assessment Unit, Institute of Zoology, Zoological Society of London, Regent's Park, London NW1 4RY, UK; 9 United Nations Environment Programme World Conservation Monitoring Centre (UNEP-WCMC), 219 Huntington Road, Cambridge CB3 0DL, UK; 10 International Union for Conservation of Nature, 28 rue Mauverney, Gland 1196, Switzerland; 11 World Agroforestry Center (ICRAF), University of the Philippines Los Baños, Laguna 4031, Philippines; 12 School of Geography and Environmental Studies, University of Tasmania, Hobart, Tasmania TAS 7001, Australia; 13 Woodley and Associates, Chelsea, Quebec J9B 1T3, Canada

## Abstract

Ensuring that protected areas (PAs) maintain the biodiversity within their boundaries is fundamental in achieving global conservation goals. Despite this objective, wildlife abundance changes in PAs are patchily documented and poorly understood. Here, we use linear mixed effect models to explore correlates of population change in 1,902 populations of birds and mammals from 447 PAs globally. On an average, we find PAs are maintaining populations of monitored birds and mammals within their boundaries. Wildlife population trends are more positive in PAs located in countries with higher development scores, and for larger-bodied species. These results suggest that active management can consistently overcome disadvantages of lower reproductive rates and more severe threats experienced by larger species of birds and mammals. The link between wildlife trends and national development shows that the social and economic conditions supporting PAs are critical for the successful maintenance of their wildlife populations.

## Abstract (web summary)

Protected areas are intended to safeguard wildlife, but their effectiveness has at times been questioned. Barnes, Craigie, and colleagues show that protected areas do offer refuge—maintaining their bird and mammal abundances—but with greater success for larger-bodied species and in more developed nations.

## Graphical abstract

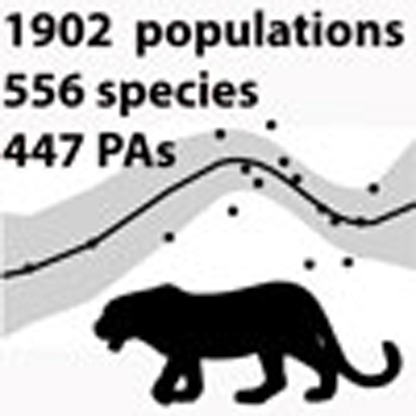

Biodiversity is in crisis^1^,^2^. A key response to global biodiversity declines^3^ and the associated threatening processes^4^, has been the establishment of protected areas (PAs). PAs underpin most global and national conservation strategies^5^, covering at least 15.4% of global land surface area^6^. The importance of PAs is set to increase further given the latest Convention on Biological Diversity targets to increase global land coverage of PAs to 17% by 2020 (ref. 7). Ensuring that biodiversity is maintained within-PA boundaries is consequently fundamental to achieving global conservation goals.

Despite a central objective of PAs being to conserve wildlife populations within their boundaries, many PAs are experiencing undesirable wildlife population declines^8^,^9^. Worldwide, wildlife population changes in PAs are patchily documented^10^, unquantified and poorly understood. While a commonly held conception is that PAs are effective at maintaining wildlife populations within their borders, this assumption has not been widely tested. Conversely, the perception that some PAs are failing, or at least performing inadequately, has precipitated calls to radically change both conservation decision-making and PA management^11^, and emphasized the need to ensure that PAs are effectively managed in the long term^12^.

It is thus vital to quantify how well PAs are conserving wildlife^13^, and to identify enabling conditions and barriers to effective conservation. By identifying properties of those PAs more likely to maintain wildlife populations, it will be possible to ensure new PAs are established in a more spatially and financially efficient configuration and maximize biodiversity outcomes within resource constraints. Without a better understanding of those factors that contribute to wildlife outcomes in PAs, then their selection, design and management are likely to remain sub-optimal.

Wildlife population change is an important and useful metric for evaluating wildlife conservation outcomes in PAs. It is sensitive to long-term environmental change^14^, often directly linked to PA objectives, and valuable in diagnosing extinction risk^15^. Critically, population trends can also quantify biodiversity change in a variety of habitat types, including savannah and other non-forested habitats, complementing studies of PA impacts on maintaining forest cover^16^. We compiled an extensive data set of 1,902 vertebrate population abundance time series from 447 terrestrial PAs, and calculated bird and mammal population trends for 556 species as a metric of PA effectiveness in meeting conservation goals. Direct counterfactual data^17^ from similar but unprotected populations were not available due to insufficient monitoring effort outside PAs. We conducted a broad-scale evaluation of within-PA wildlife abundance trends (change over time) to identify properties of PAs that contribute to variation in trends among PAs. Learning from conservation outcomes for wildlife among PAs has the capacity to dramatically improve policy and management for native wildlife in PAs by promoting the propagation of enabling practices globally.

We find that on average PAs are maintaining the abundance of populations of monitored birds and mammals within their boundaries, and that wildlife population trends are more positive in countries with higher development scores, as well as for larger bodied species. This body mass finding suggests active management can overcome disadvantages of more severe threats experienced by larger species^18^,^19^. It also suggests there is a need to manage smaller species directly, rather than assuming that management actions targeted at conservation of iconic taxa will lead to effective conservation of all species. Our results also underscore the need to address social and economic conditions that support PA management—as these appear to be critical for maintaining wildlife populations within PA borders.

## Results

### Dataset

**Figure 1:**
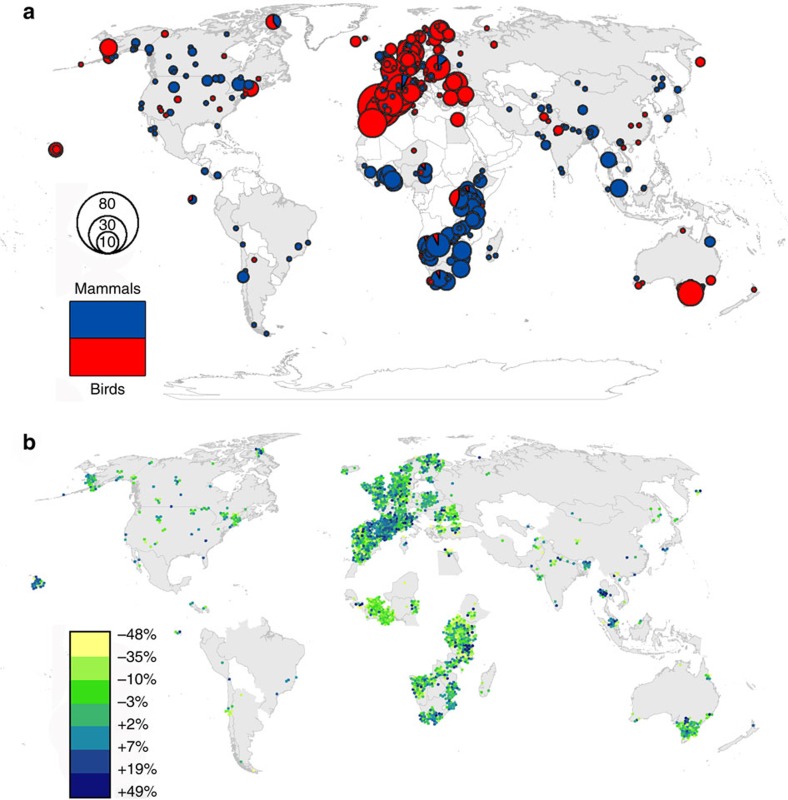
Locations of our PA population time series. Countries in grey are those included in the analysis. (**a**) Proportionally sized pie charts indicate the number of bird (red) and mammal (blue) time series in each PA. (**b**) Mean per cent annual change in population abundances for each PA represented. Lighter (more yellow) dots represent greatest declines, and darker (more blue) dots greatest increases.

The full data set contains 1,902 population time series for 556 species in 447 PAs (Supplementary Table 1, Supplementary Data 1 and 2) across 72 countries (Fig. 1 and Supplementary Fig.1), from time periods between 1970 and 2010. The species in our data set are dominated by large mammalian herbivores and waterfowl (body mass distributions—Supplementary Fig. 2), reflecting taxonomically uneven global monitoring efforts. Note our data end before the recent poaching crisis that has significantly reduced populations of African elephants *(Loxodonta africana*) and also affected rhinoceros species (*Diceros bicornis* and *Cerotherium simium*)^20^.

### Overall Trends

**Figure 2:**
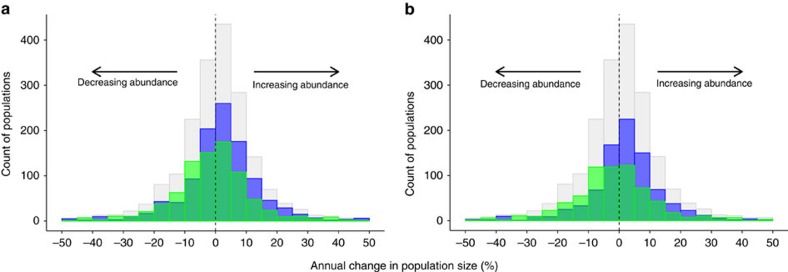
Frequency distribution of wildlife abundance changes in protected areas. (**a**) Changes by species type. (**b**) Changes by location. In **a**, grey shows all species, green shows all mammals and blue shows all birds. In **b**, grey shows all sample populations, green shows sampled populations in Africa and blue shows sampled populations in Europe.

Overall, the mean percentage annual change in population size within PAs was near zero (slightly positive: mean 0.52%, median 0.81%, s.d. 12.7, Fig. 2). Overall, bird trends were marginally positive (mean annual change 1.72%, median 1.71%, s.d. 12.45, Table 1), whereas mammal trends were slightly negative (mean −1.00%, median −0.62%, s.d. 12.45, Fig. 2a). Trends in Europe were more positive than those in Africa (Fig. 2b and Table 1).

Population trends showed substantial variation across species and PAs (Fig. 1b). To explore this variation, we tested factors previously identified as likely to influence biodiversity outcomes in PAs^10^, and collated data sets addressing those factors (Supplementary Tables 2–4). We examined six groups of possible influences: (1) PA design (for example, size, shape, IUCN management category), (2) socio-economic context of the region and country in which the PA is located (for example, wealth, corruption), (3) species' traits which might determine response (for example, body mass), (4) local human impacts (for example, road density, land-use change), (5) biophysical context (for example, PA elevation) and (6) time series characteristics (for example, length). We used linear mixed effect models to account for the hierarchically nested data structure, and in addition to a global model, produced separate models for mammals and birds, and for the two most data-rich regions (Europe and Africa).

### Model Results

**Figure 3:**
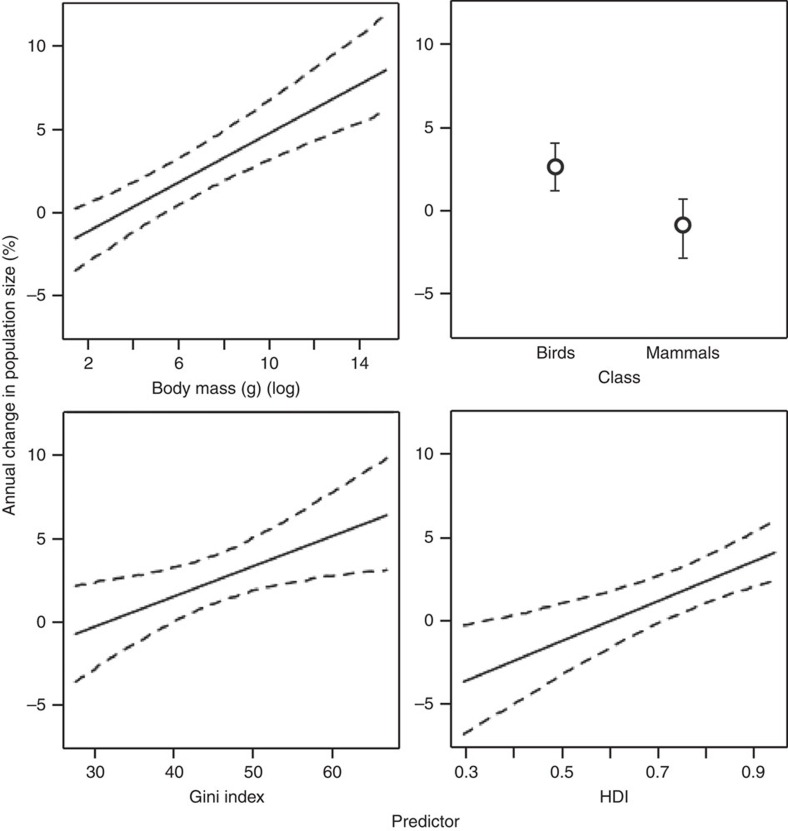
Partial-effects plots for variables in the most parsimonious global model. Partial-effects plots showing fitted relationships between change in population size and (**a**) body mass, (**b**) taxonomic class, (**c**) Gini index and (**d**) Human Development Index (HDI). In **a**, **c**, and **d**, dashed lines are 95% credibility intervals. In **b**, the circles indicate the estimated partial effect size for each factor level with credible intervals displayed as error bars.

**Figure 4:**
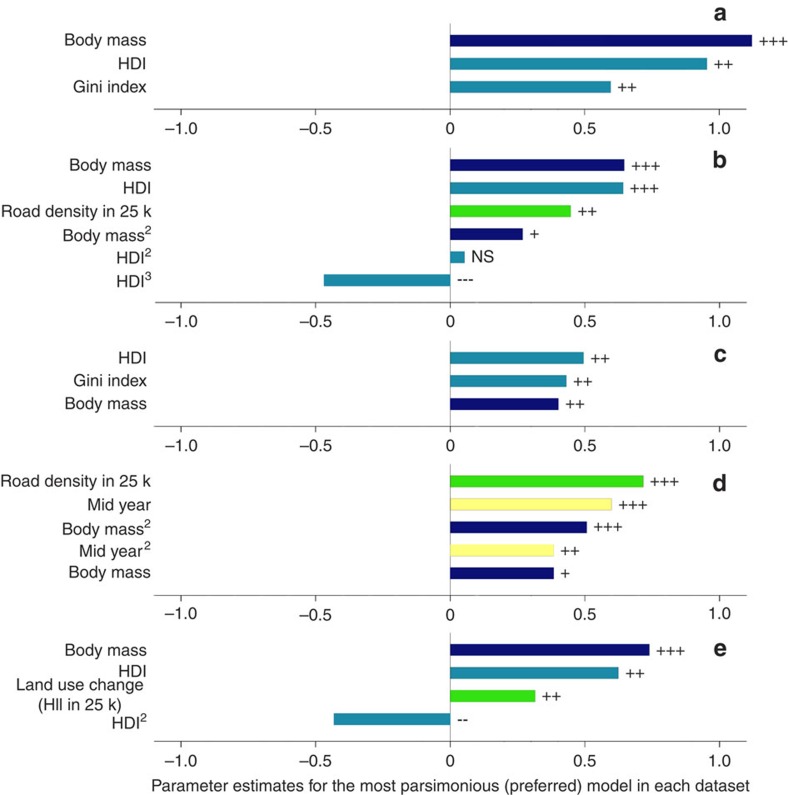
Informative continuous fixed effects in preferred models for each subset. Data are shown for (**a**) global, (**b**) mammals, (**c**) birds, (**d**) Africa, (**e**) Europe. Bar lengths indicate the size of the parameter estimates and their explanatory power in the model. Significance levels derived from highest posterior credibility intervals from Markov-Chain Monte Carlo methods: NS *P*⩾0.05;+*P*≤0.05; ++*P*≤0.01; +++*P*≤0.001. Colours show groups of potential explanatory factors: dark blue, species' traits; light blue, socio-economic context; green, local human impacts; and yellow, time series characteristics. Superscripts indicate power (that is, squared, cubed) of the variable for those variables which exhibit higher order relationships.

**Figure 5:**
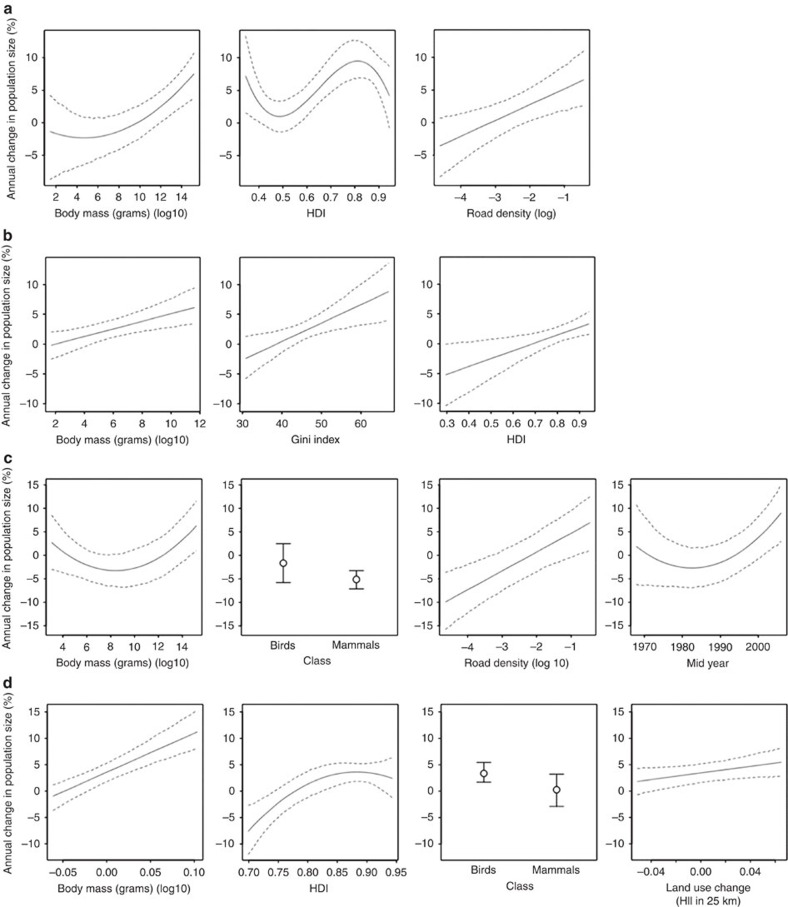
Partial-effects plots for the most parsimonious model of each subset modelled. Data are shown for (**a**) mammals, (**b**) birds, (**c**) Africa, (**d**) Europe. Dashed lines are 95% credibility intervals based on MCMC sampling with 10,000 samples. They show the relationship between each fixed effect: (**a**) body mass, HDI, road density; (**b**) body mass, Gini index, HDI; (**c**) body mass, class, road density, mid year; and (**d**) body mass, HDI, class, land-use change (Human Impact Index (HII) in 25 km), and population trends when all others are held constant. For categorical variables (class) dots indicate the estimated partial effect sizes of each factor level, and error bars show 95% credible intervals. HDI, Human Development Index; MCMC, Markov-Chain Monte Carlo.

Correlates of species population trends in PAs were identified across four of the six groups of factors (Figs 3,4,5, Supplementary Fig. 3 and Supplementary Tables 5 and 6). In order of importance: first, in most models, population trends were more positive in areas with higher national Human Development Index scores (global, mammal, bird and Europe models), and greater Gini indices (that is, level of income inequality, in global and bird models; see also Supplementary Note 1). Second, population trends increased with body mass (in all models) and differed among taxonomic classes (all models where tested, except Europe). Third, among our measures of anthropogenic impact, population trends were positively correlated with local road density (mammal and Africa models) and local Human Influence Index (Europe model). Fourth, population trends in African PAs were more positive in later years. Neither of the other two groups of factors, PA design or biophysical context, emerged as significant fixed effects in any models: in all models species and socio-economic factors were more important. Models were found to be robust to the effects of phylogenetic influence as reflected by taxonomy, and exhibited low sensitivity to statistical outliers such as the positive outliers of African elephants and rhinoceroses. Note our abundance data pre-dates the post-2008 surge in illegal hunting of elephants and rhinoceroses^20^,^21^.

## Discussion

Given the central role of PAs as global conservation tools, it is reassuring that, on average, monitored populations within PAs are stable or marginally positive (mean 0.52% annual increase). The differences between birds and mammals (birds more positive), and between Europe and Africa (Europe more positive) are likely an effect of differing regional histories, as well as current pressures. Large-scale land conversion outside the tropics has led to broad-scale historical extirpations^22^. More recent policy changes in Europe have led to widespread improvements in biodiversity management^23^, and the recovery and reintroduction of many wildlife populations, including birds in PAs^24^. African wildlife populations are typically more intact in absolute terms, but are under increasing anthropogenic pressure; causing abundance declines^8^.

Strikingly, larger-bodied species had more positive population trends in all models except Europe, indicating that PAs are more likely to maintain populations of larger-bodied wildlife than smaller bodied species. This finding is consistent across geographic realms, and taxonomic class, so it was not driven by the difference in body mass between birds and mammals. In the mammal and Africa models the relationship of body mass to trend was u-shaped (Fig. 5), suggesting perhaps that the smallest species are more resilient because of their high reproductive rates^25^; while intermediate sized species, lacking active management and having slower reproductive rates, are experiencing greatest decline. These findings have not previously been quantitatively demonstrated, but are supported by previous anecdotal evidence from Kenyan PAs^26^, which posited a shift from elephant and rhino to poaching smaller species in response to increased penalties, before the recent ivory and horn poaching crisis.

One explanation for these findings is that threat processes of high severity (for example, hunting) are impacting intermediate bodied species particularly badly—a pattern previously detected among threat processes for mammals and birds^27^,^28^,^29^. Management effort and external project funding is commonly prioritized towards large-bodied flagship and charismatic species^30^, and larger species are key to tourist revenues and public/political priorities^31^. As a result, monitoring and management actions focus on the needs of these species and it is likely that smaller species do not receive the same benefits from PAs. We detail interactions between body mass, threatening processes and influential factors in a conceptual diagram (Supplementary Fig. 4). Larger species also tend to be preferred for ecological study and monitoring. Consequently, population declines are likely to be noticed sooner and their causes better understood, leading to more effective management responses. This result has substantial implications for future allocation of conservation effort among species within PAs.

Indicators of greater human wealth (gross domestic product) and development (Human Development Index) were associated with more positive population trends. Wealth and development have been shown to have a complex relationship with conservation. Poor outcomes have been associated with both historical and accelerating threatening processes, while increasing wildlife trends can be associated with greater awareness and management capacity, that is, wealthier countries may have more resources available for PA management, and spend more on conservation^32^,^33^,^34^. Moreover, human populations in wealthier areas have less need for resources directly extracted from PAs to support livelihoods (for example, bushmeat, firewood). A final contributing factor to this pattern could be historical species loss (an extinction filter effect)—with the biota of wealthier countries having been more extensively purged of species which are more sensitive to anthropogenic threats^35^.

This finding of an association with development is promising, as it shows that additional capacity or decreased dependence on natural resources, can help ameliorate wildlife declines in PAs. Thus, economic development and the associated improvement in food security and governance may lead to effective conservation. In reality, outcomes are likely related to a balance of multiple socio-economic factors. Regardless of which of these explanations is dominant, extra effort will be required to retain species in developing regions. However, to avoid the extinction filter in developing regions resulting in wildlife loss catching up with historic losses in developed countries, extra effort will be required to retain species in these countries. The marginal finding that higher national Gini indices predict more positive population trends is unexpected. The result can be explained by a small number of countries (for example, South Africa) that have both very high Gini indices and relatively positive wildlife trends.

Counter-intuitively, more positive trends in PAs were correlated with increased anthropogenic landscape changes, indicated by locally denser road networks in the mammals and Africa models, and land-use change in the Europe model. Development of new roads may open up areas and cause wildlife and habitat declines^36^, but in areas of historical road construction there may be a filter effect with PAs currently experiencing a wildlife recovery, especially for certain robust species^37^. Wildlife populations surrounded by extensive historical land clearing and roads perhaps experienced their declines before our data set but are now stable, whereas places with fewer roads have recently experienced development and clearing, driving declines^38^. Conversely, higher road density may correlate with increased levels of resources for management, improving PA outcomes: road density was found to be positively correlated with greater PA management effectiveness by Geldmann *et al*.^37^

Several well-studied ecological factors that conservation theory predicts should be important determinants of wildlife trends, including PA size^39^ and PA shape^40^, have no explanatory power in our models. We do not interpret the lack of significance of these variables to mean they are not important. We suggest that over the timescales addressed by our data their influence is overwhelmed by either the priorities of managers, the more general landscape scale recovery of large species^23^, or the socio-economic context of PAs, but we cannot not easily differentiate between these drivers. Over multi-decadal timescales the ecological drivers are still likely to be influential, but our results show the importance of managing PAs for more immediate threats. A substantial quantity of research effort examines optimal design of PAs and PA networks with respect to ecological processes, but our findings suggest that greater conservation benefit would result from a focus on optimizing network design considering management and human influences on PAs.

The ability of PAs to maintain species populations is critical to global conservation. Our finding that abundances of monitored birds and mammals are maintained within PA boundaries is encouraging. However, our results show that PAs do not work equally well for all species or in all circumstances. Moreover the recent poaching crisis in Africa shows that population gains can be rapidly reversed, if the threatening processes grow too large to mitigate^20^, emphasizing the need to scale management effort with threat intensity^41^,^42^. In addition, the time series data for this analysis are from PAs that are older, larger, and further removed from humans than most PAs (see Supplementary Fig. 5). Hence, we should not be complacent. These findings are likely to represent a best-case scenario for protected populations as they are sourced from PAs experiencing lower than average anthropogenic threat. If we expect PAs to act as refuges for all species in perpetuity, then a wider range of species must be targeted for management, and a particular focus on conserving medium-sized species may be required.

Further, it is clear that PAs do not exist in a vacuum. Our results show that the social and economic conditions that support PA management are critical for the maintenance of wildlife populations within PA boundaries. Anthropogenic drivers of wildlife abundance change appear to have more influence than those drivers affecting ecological processes such as PA size, at least over the period of a few decades. Much of the research effort into PAs targets ecological processes but it seems greater return would come from focusing efforts more on anthropogenic drivers of PA performance.

Managing PAs' socio-political, rather than simply ecological, dimensions is pivotal to wildlife conservation in PAs. Human dependence on PA resources in poorer countries must be addressed if existing PAs are to retain their contents as these nations continue to develop. Finally, to understand the return on our investment in PAs in the long-term, best practice adaptive management and systematic monitoring of biological outcomes, including appropriate counterfactual monitoring, is essential^43^. The tools to understand impact and improve outcomes exist—but we must strengthen the will and capacity to implement them.

## Methods

### Wildlife population time series data

A global database of population abundance time series of all available time series for native birds and mammals in terrestrial PAs worldwide was compiled from sources including the Living Planet Database^44^, PA agencies, published literature, grey literature and non-governmental organizations. Time series in the data set represent the majority of the data available globally to address this topic. Time series consisted of population abundance count estimates, or proxies of abundance such as nest density, mark-recapture or density estimates. Marine species, other than those with at least one critical life history stage on land (for example, breeding colonies), were excluded. We used population time series that met a number of criteria based on Collen *et al*.^44^: (1) the technique used to measure abundance was comparable over the length of the time series; (2) the geographic location of the population was provided; (3) the majority (>50%) of the measured population was within a PA; and (4) population time series were a minimum of 5 years in length between 1970 and 2010, with at least three measures of abundance within that time period (that is, an estimate was not required for every year within a time series). Where data were available for multiple time points in a single year (for example, wet season and dry season), data were standardised to obtain a single per annum abundance estimate for each population. Standardization was carried out using the most appropriate and comparable method given data type and species ecology to obtain an estimate of species population changes most likely to remain consistent through time, and accurately represent population change, in accordance with established LPI database practice^44^,^45^. For instance, monthly abundance counts were averaged (using mean estimates) for resident non-irruptive species.

### Estimation of wildlife population trends

Population trends were estimated by fitting a generalized linear regression model on time with a log-link function to each population time series^46^,^47^. The trend was taken to be the slope value from each regression. Fitting a log-link function assumes the response variable (population abundance) has a Poisson error distribution, and that the logarithm of its expected value can be modelled by a linear combination of unknown parameters, and is most appropriate for zero-inflated data, such as count and catch per-unit effort data^46^. Leading and trailing zero values (that is, zeros which occurred at the beginning or end of time series) were excluded from each time series before calculating trends; such zero values generally occur when populations are present at a level below that at which the sampling method is able detect individuals, rather than when a population has been extirpated or introduced. These zeros are therefore inaccurate estimates of abundance, yet they exert undue influence on the estimated slope values (population trends). Slope values exceeding ±0.5 on the natural log scale were excluded following inspection of the data distribution. Such values are equivalent to annual rates of population growth and decline of more than 50% per annum and biologically implausible over periods greater than 5 years (equivalent of a population of 1,000 individuals increasing to 7,594 in 5 years or 57,665 in 10 years). Such values are likely the result of errors in surveys or data entry^48^,^49^,^50^.

### Explanatory variable selection and preparation

Variables were selected to represent characteristics of PAs considered most likely to be important determinants in maintaining wildlife populations in PAs for vertebrate species, based on both theoretical supposition and empirical observation as identified via a comprehensive literature review^10^ and can be regarded as belonging to six groups of possible influences: PA design (for example, size, shape, IUCN management category), socio-economic context of the region and country in which the PA is located (for example, wealth, corruption), species' traits which might determine response (for example, body mass), local human impacts (for example, road density, land-use change), biophysical context (for example, PA elevation), and time series characteristics (for example, length). Several variables suggested by the literature to be important for determining PA outcomes at the site scale (for example, PA-specific management budgets, threat intensity) were unavailable for most sites, and therefore could not be tested in this analysis. The key socio-economic variables used in the analysis were only consistently available at the national scale, not at finer spatial scales relative to the PAs. A table summarising the justification for each variable*, a priori* hypotheses, and references underpinning the selection of each factor are provided in Supplementary Table 2. For each explanatory variable, information was collected at a scale based on a combination of availability, standard practice and relevance for population dynamics. Generally, this meant that the finest resolution data available at a global scale was used. Descriptions of the explanatory variables and data sources and resolution are given in Supplementary Table 3.

Spatial data were analysed using ArcGIS 10.0 and R 2.15.0 (ref. 51) using the packages raster^52^, geosphere^53^, maptools^54^, rgeos^55^, rgdal^56^ and sp^57^. PA boundaries were calculated using spatial information from the World Database of Protected Areas^58^. Spatial data relating to PA context (for example, human population density) were calculated in buffers of three different sizes (5, 10 and 25 km) around each PA polygon. Multiple buffer sizes were used because it was not known *a priori* over what distance potential correlates would be most likely to be acting. PAs represented only by point information in the WDPA were included in the analysis by creating appropriately sized circular buffers using the ArcGIS buffer tool under an equal-area (Mollewide) projection^59^. The size of the buffer was given by the area (km^2^) recorded for the individual PAs in the WDPA. In cases where the projection of the PA data set differed from the explanatory data layer, the PAs were reprojected using ArcGIS. Details of the preparation of the individual explanatory variables are provided in Supplementary Tables 3 and 4. Several explanatory data sets were available as raster layers. The R raster package^52^ was used to overlay the PA polygon onto those cells and calculate metrics based on the underlying raster cell values. However, using the raster package, overlayed polygons must cover the centre of a raster cell to be considered as inside the polygon. Small, spatially complex PA polygons may only encompass a few raster cells, particularly when overlayed on raster layers with coarse spatial resolution. The selected cells may therefore poorly represent the overall cell area truly overlapped by the polygon. For this reason, we disaggregated each raster layer by a factor of 10 (30 for the spatially coarse agricultural suitability layers) using the R raster package for small PAs (<2,000 cells overlapped by the PA polygon). We also enforced a minimum overlapping area for very small PAs before performing all calculations (noted in Supplementary Table 3 in terms of the equivalent number of raster cells before disaggregation). For some variables (noted in Supplementary Table 3), PA polygons were clipped of adjoining marine areas in ArcGIS using the World Vector Shoreline Plus layer (http://shoreline.noaa.gov/data/datasheets/wvs.html).

Explanatory variables were transformed to normalize distributions, where necessary, and standardised using the R function ‘scale' so all variables in the data set had equal means and standard deviations but different ranges. Normalized and standardised variables were evaluated to determine collinearity by visual inspection of the data and by calculating Pearson's correlation coefficient (see Supplementary Fig. 6).

### Population trend modelling relative to explanatory variables

The slope of the generalized linear regressions for all populations was used as the response variable to address the question: what factors predict trend in abundance for bird and mammal species in terrestrial PAs? We applied a linear mixed-effects modelling approach to explore the key correlates of wildlife population of birds and mammals through time in PAs. Explanatory variables were hierarchically spatially structured; at the species, PA and national levels. We used linear mixed-effects modelling to account for the data structure and investigate the relationship between population trends and the suite of potential explanatory variables. Mixed-effects models allow partitioning of the variance in population trends in a nested hierarchy^60^,^61^. In this analysis, the data were structured such that each population trend referred to a particular species within a given PA. Most sites contain several species, and most species occur at several sites so there are multiple observations of individual species across a suite of site subsets, such that individual population trends are not mutually independent. Further, populations were distributed non-randomly across continents and countries. All models were implemented in R 2.15.0 (ref. 51) using the packages lme4 (ref. 62) and MuMin^63^. Random effects were kept consistent in all models with species, site (PA), and country fitted as random effects in every model based on the a priori understanding of the data structure. In models where both birds and mammals were present, taxonomic Class was included as a fixed effect in the models. Models were generated for the entire global data set and subsets for taxonomic class and geographic realm. Subset models were generated for: birds, mammals, Africa and Europe. Although some population data was available outside Europe and Africa, other geographic realms had insufficient sample sizes to construct subset models.

### Modelling procedure

Model selection was made following an information-theoretic approach, using the corrected Akaike Information Criterion AICc^46^,^64^. Forward and backward stepwise model selections were conducted to narrow the set of candidate models. Thereafter all possible subsets of candidate models were compared using the function dredge^63^. This included testing all plausible interactions, and polynomials (orthogonal squares and cubes). Some variables could not be fitted simultaneously as they were highly collinear (Pearson's correlation coefficient >0.5). In these cases the variable with the greatest explanatory power, as defined by the best AICc value, was found by substitution. Substitution was conducted by exchanging variables in the model that were collinear and expected to explain the same component of the variation to assess which of them provided the best fit to the data. Fit was assessed by change in AICc, and during substitution the remainder of the model specification was held constant. Substitution was carried out both during initial data exploration and before final model selection for each data set.

For each model with a ΔAICc of less than four the fitted residuals were examined using qq-plots, Cook's distance leverage plots and histograms. Models selection was tested for sensitivity to outliers through systematic removal and replacement of outlying data. Outliers were identified using qqplots and Cooks distance leverage plots of the model residuals. When outliers were removed the preferred models and their parameter estimates were remarkably consistent, and additionally exhibited low sensitivity the removal of extremely large species such as Africa Elephant (*L. africana*) and rhinoceroses (*D. bicornis* and *C. simium*). The elephant and rhino outliers were highly positive; if data including the recent years' poaching-related population declines of these species had been available these species may not have been outliers. Fitting genus and order as random effects, to test for phylogenetic influence as reflected by taxonomy, did not improve model fit significantly for any models. When they were fitted, body mass parameter estimates remained stable and significant.

### Effect sizes

Markov-Chain Monte Carlo Highest Posterior Density estimates with 10,000 samples were calculated to estimate effect sizes and 95% credibility intervals^46^ for the most parsimonious models. Partial-effects sizes were calculated and plots produced for parameters of the best-fit models (Fig. 5). Variable relative importance was calculated following Zuur *et al*.^65^ using Akaike weights and relative frequency standardised across all models with a ΔAICc <4 (Supplementary Fig. 3).

### Data availability

All relevant data are available from the authors upon request. The population time series are available from the Living Planet Database to registered users: http://www.livingplanetindex.org/data_portal. Links and citations for all the freely available predictor data sets are available in Supplementary Table 3.

## Additional information

**How to cite this article:** Barnes, M. D., Craigie, I. D. *et al*. Wildlife population trends in protected areas predicted by national socio-economic metrics and body size. *Nat. Commun.* 7:12747 doi: 10.1038/ncomms12747 (2016).

## Supplementary Material

Supplementary Information Supplementary Figures 1-6, Supplementary Tables 1-6, Supplementary Note 1, Supplementary References

Supplementary Dataset 1 Table listing all species included in the analysis

Supplementary Dataset 2 Table listing all protected areas included in the analysis

## Figures and Tables

**Table 1:** Annual percentage population change in each data set.

**Data set**	**Mean**	**Median**	**s.d.**
Global	0.52	0.81	12.72
Mammal	−1.00	−0.62	12.45
Bird	1.72	1.71	12.45
Africa	−1.79	−1.67	13.68
Europe	2.05	2.15	12.05
