# Supplementary material for: Wildlife population trends in protected areas predicted by national socio-economic metrics and body size
Source: Nat Commun. 2016 Sep 1;7:12747. doi: 10.1038/ncomms12747 (PMC5025815; doi:10.1038/ncomms12747)
Supplement: Supplementary Information — Supplementary Figures 1-6, Supplementary Tables 1-6, Supplementary Note 1, Supplementary References [file ncomms12747-s1.pdf]

Supplementary Figures

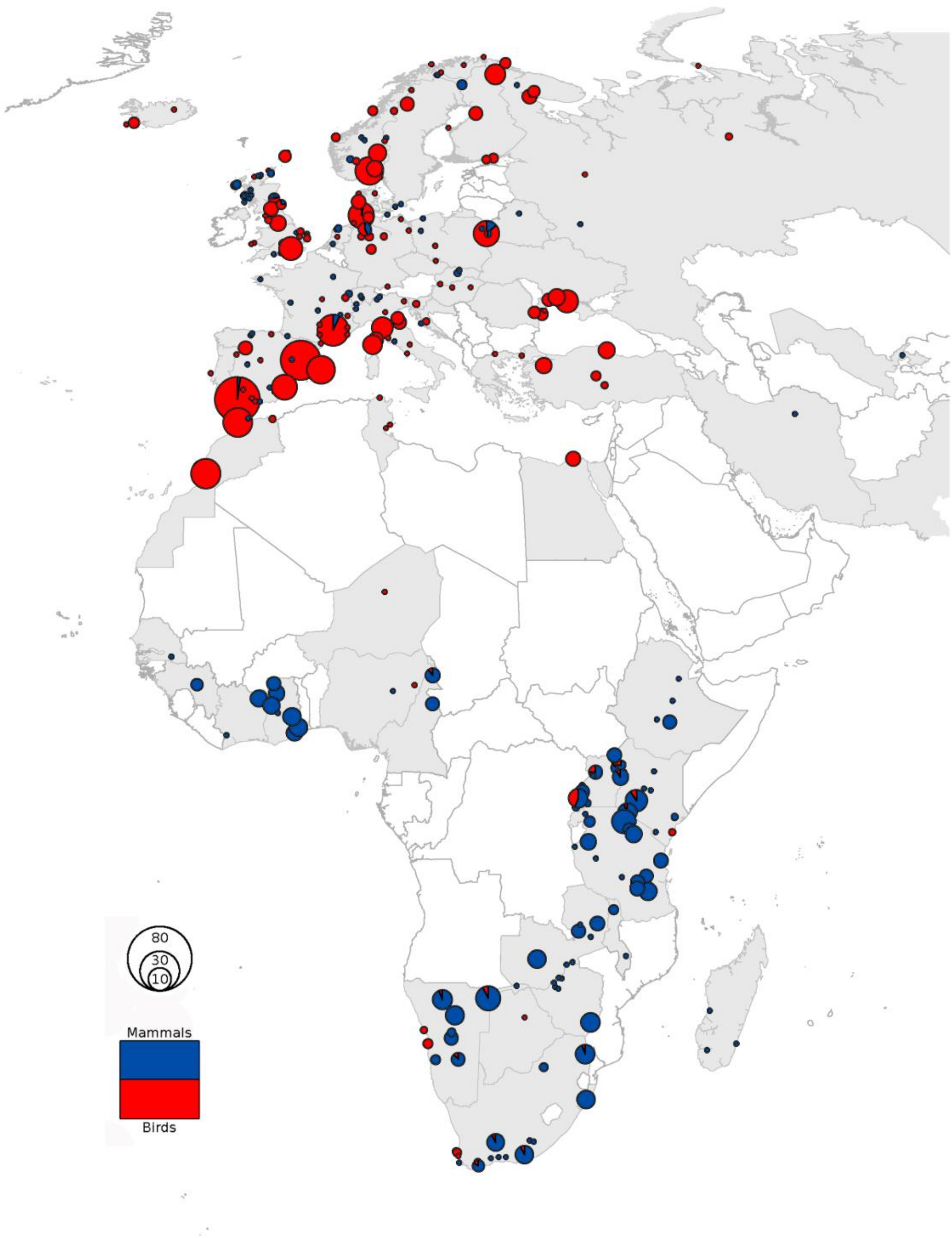

Supplementary Figure 1. Locations of PA population time series in Africa and Europe

Enlargement of data rich regions of Figure 1a. Countries in grey are those included in the analysis.

Proportionally sized pie charts indicate the number of bird (red) and mammal (blue) time series in each PA.

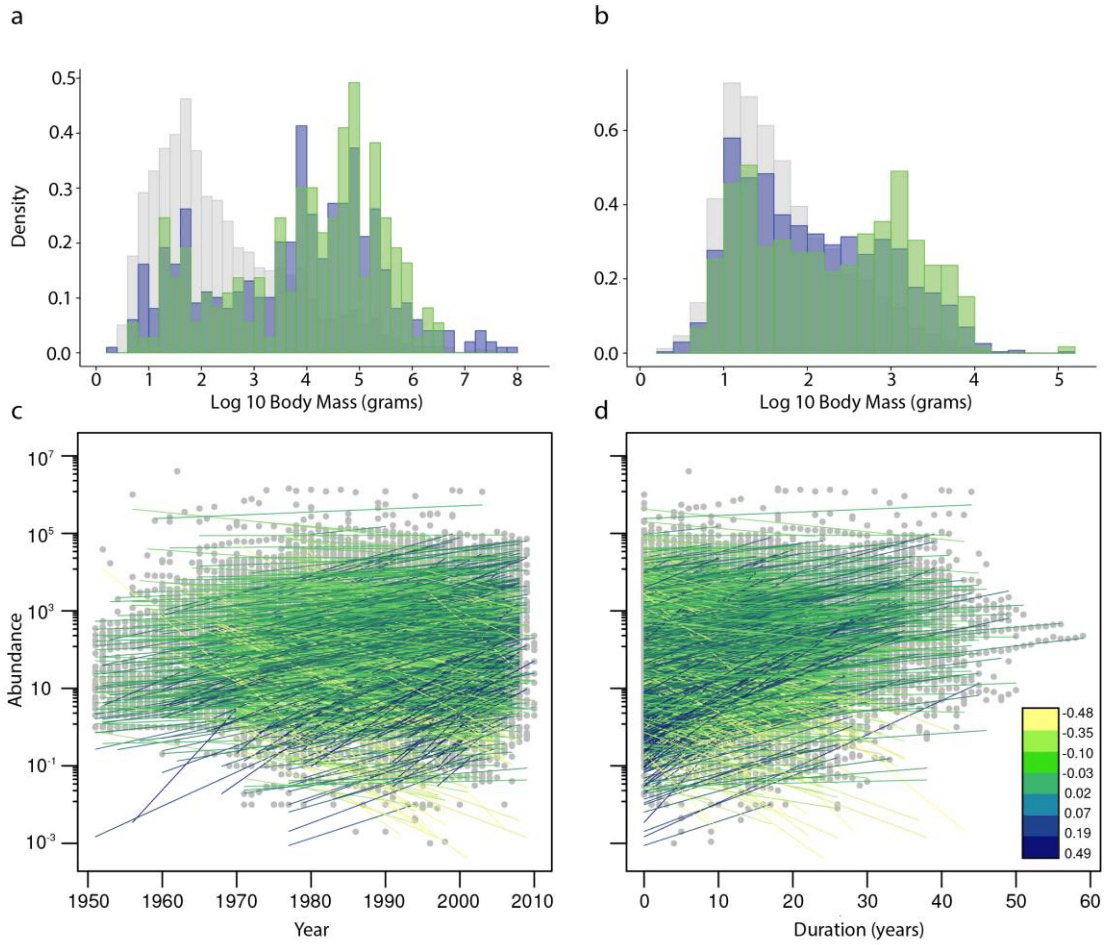

Supplementary Figure 2. Density histograms comparing distributions of body mass for (a) mammals (b) birds Within each class, grey bars indicate global distribution of body mass (n_{birds} =8610 spp; $n_{mammals}$ = 5416 spp), blue bars indicate the body mass distribution of all species in the LPI database[6] ($n_{mammals}$ = 497, n_{birds} = 1355), and green bars indicate the body mass distribution for species in this study ($n_{mammals}$ = 208, n_{birds} = 348). Bird body masses were obtained from the CRC handbook (Dunning 2008) and mammal body masses from PanTHERIA[27]. The interquartile range in this study is 17.2 – 263kg for mammals and 63.4 – 292g for birds. In contrast, the interquartile range of all terrestrial mammals is 1.14- 3.25kg [1] from shrews (1-3g) to elephants (~1500kg); and all birds is 16-158g [2], from hummingbirds (2-5g) to ostriches (~110kg); **c, d,** Population time series fitted with general linear models for **(c)** Temporal change in population abundance and **(d)** temporal change plotted from a uniform time point for all populations. Data points are represented by grey circles and models fitted by solid lines. Each line corresponds to a modelled population with colours indicating the slope of the trend.

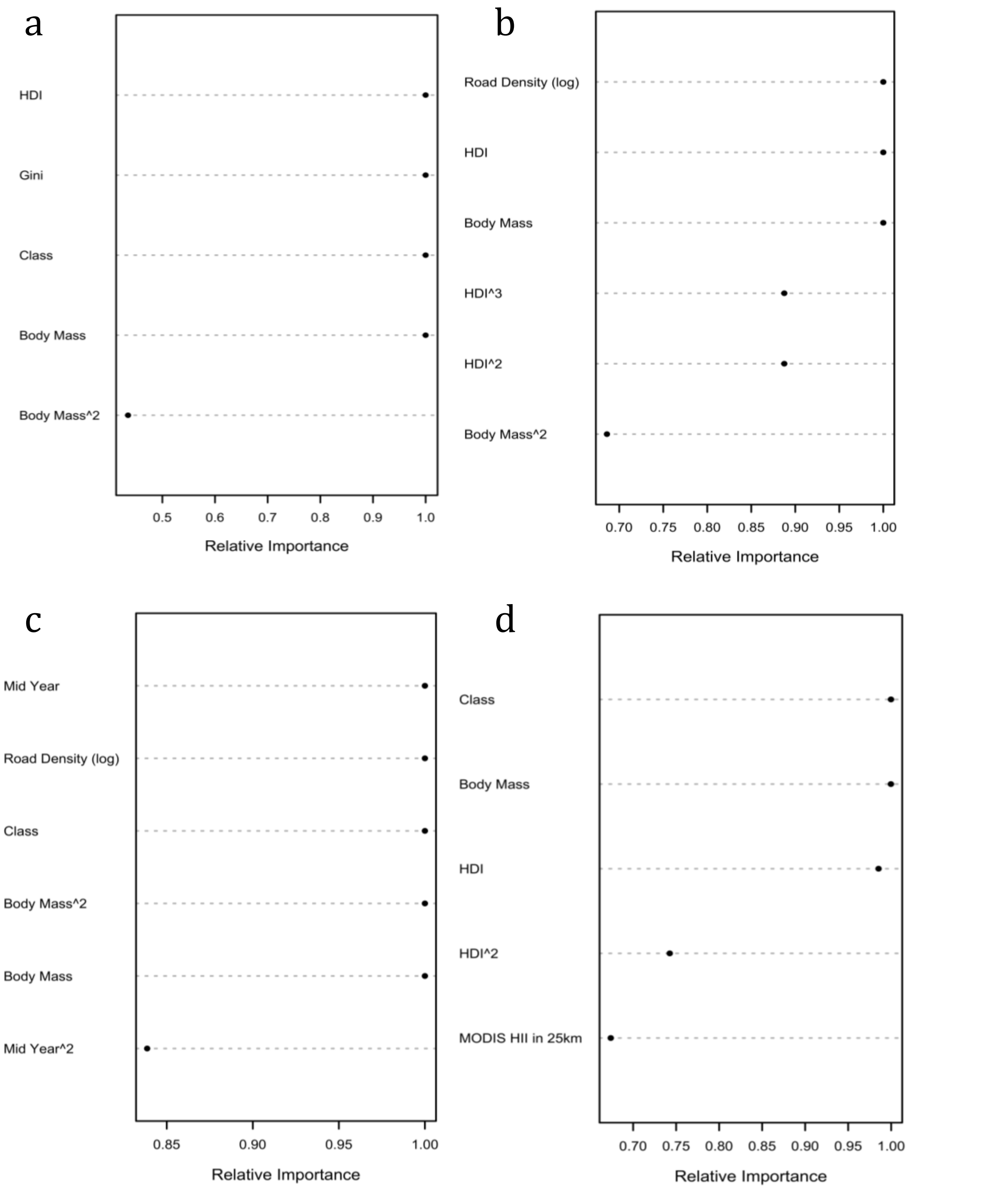

Supplementary Figure 3. Model variable relative importance plots

Dotplots illustrating the relative importance of each explanatory variable in the set of models with ΔAIC <4 compared to the best model from the **(a)** global dataset, **(b)** mammal subset, **(c)** Africa subset and **(d)** Europe subset. Relative importance calculated using rescaled Akaike weights (w_i), and varies between zero (not informative) and one (essential). No plot was drawn for the bird subset due to having only one candidate model with ΔAIC <4.

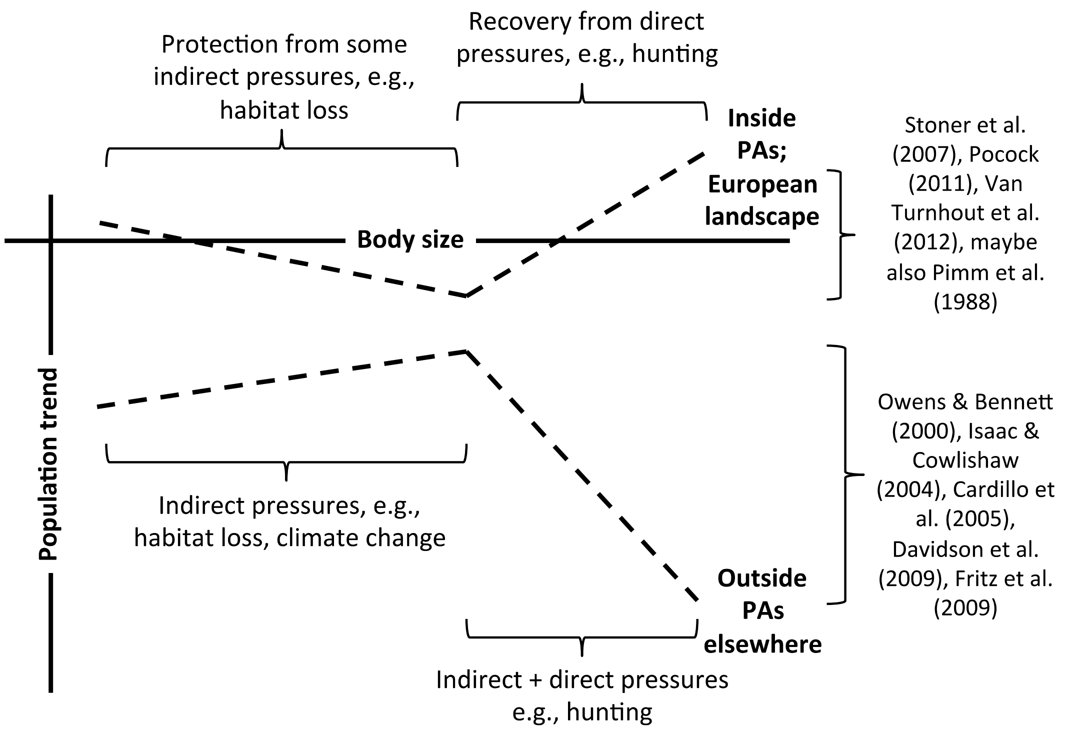

Supplementary Figure 4. Conceptual model

The diagram depicts the relationship between body mass and population trends with associated potential drivers. The unexpected positive correlation of body size and positive population trends contrasts with the common pattern of greater body size usually correlating with increased extinction risk [3,4]. However, extinction risk varies widely across clades of mammals [3,5-7] and birds [8], and body mass interacts with threatening processes [8-10]. Alternately, large bodied species could conceivably be increasing everywhere, including in PAs as a result of broad-scale policy initiatives e.g. Bans on DDT use, egg collection, and hunting in Europe and North America [11,12].

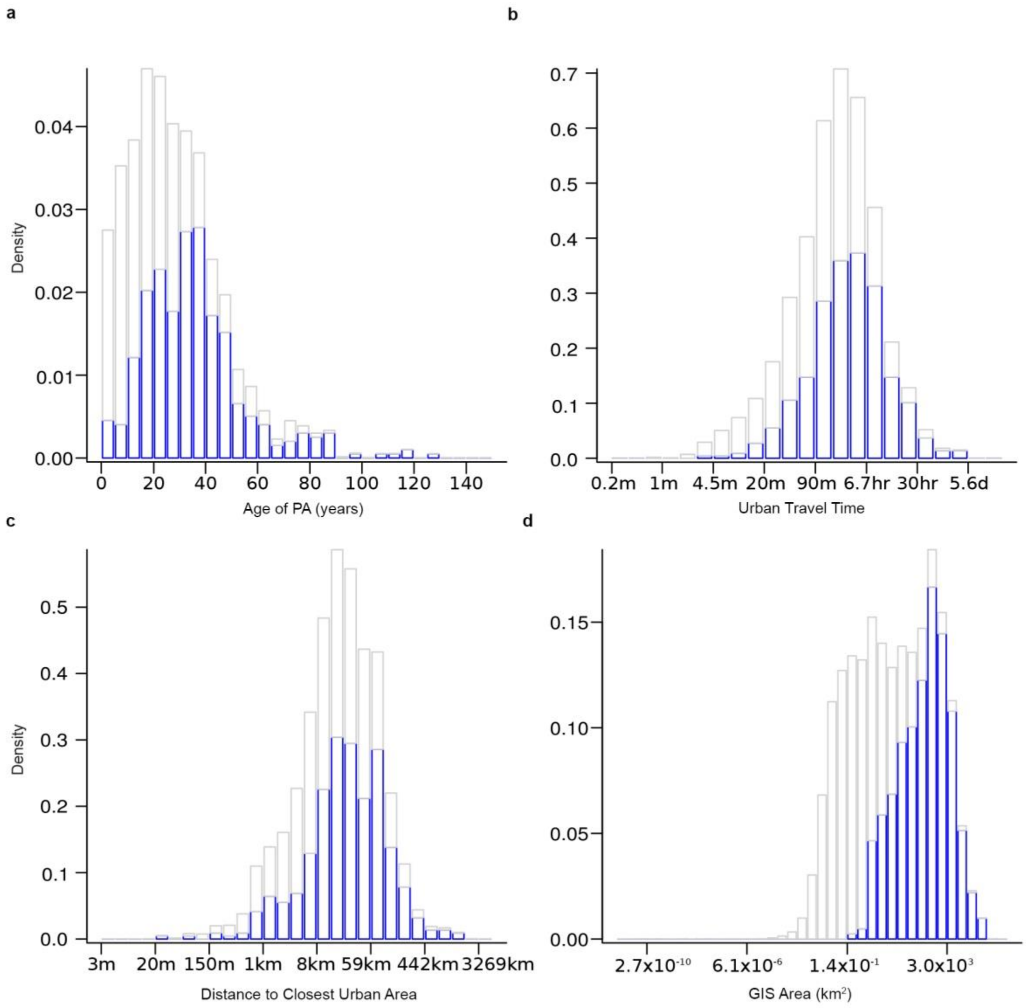

Supplementary Figure 5. Comparison of characteristics between sampled PAs and all PAs

Comparative histograms illustrating the density across the distribution of values for all protected areas in the world database of protected areas (WDPA; grey outline) and the density across the distribution of values in our dataset (blue outline) (a) Protected area Age (b) Urban Travel Time (c) Distance to closest urban area (d) Area of the protected area

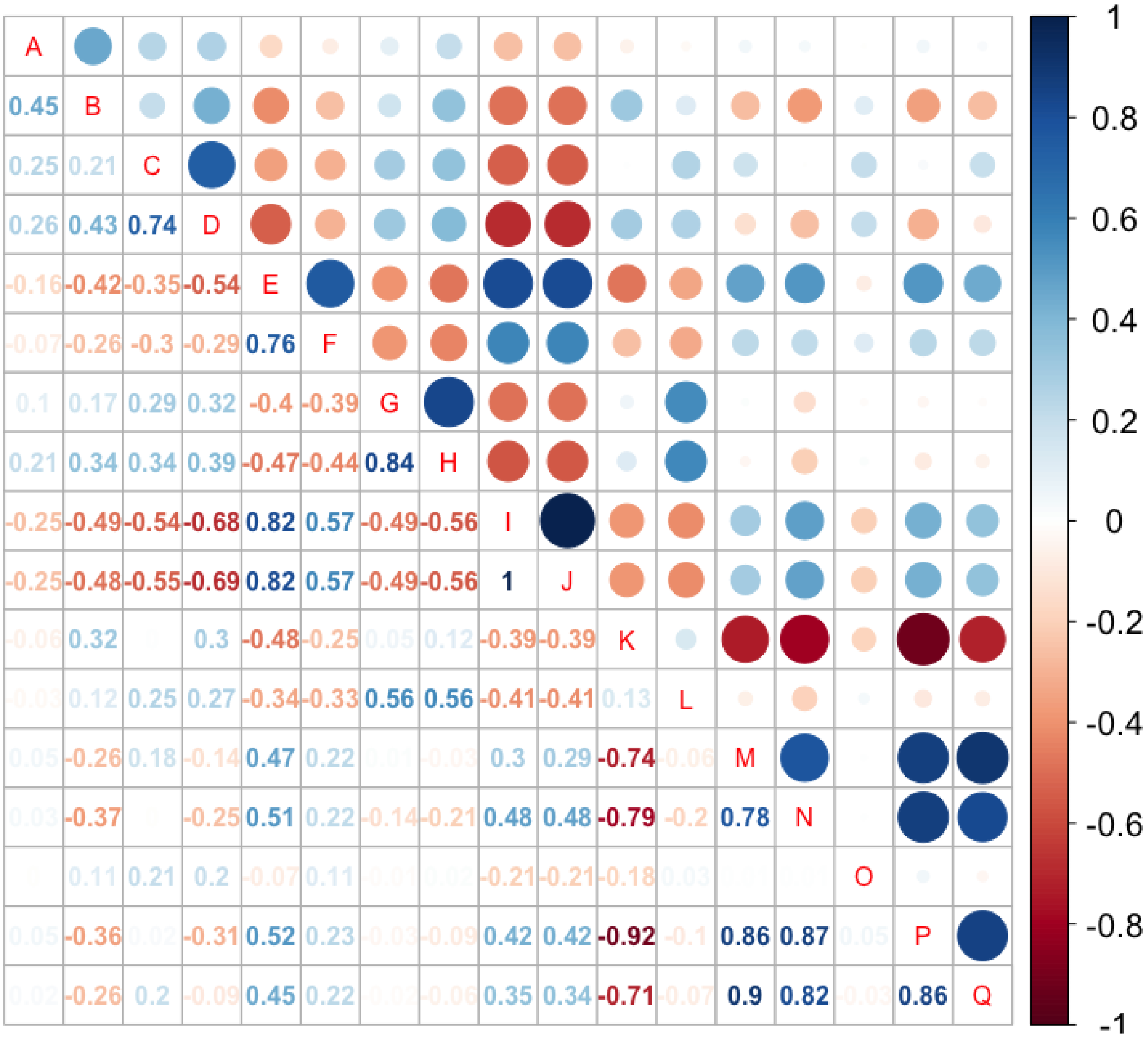

Supplementary Figure 6. Pearson's correlation coefficients of the key explanatory variables used in the models Colours of text and circles indicate negative (red) and positive (blue) correlations between individual variable pairs. Circles and text show the same data; circle size illustrates the strength of correlation. Key to variables – A: Slope (SRTM90MEANSLOPE), B: Elevation (SRTM90MEANELEV), C: Percentage Natural Land-cover in Buffer from GLOBCover (Nat_%_LC), D: Percentage Natural Land-cover in Buffer from MODIS (Nat_%_LC), E: Road Length (gROADS), F: Road Length (RRT), G: Urban distance (UrbanDist), H: Travel time to urban areas (UrbanTravel), I: Human Influence Index (HII), J: Human Footprint Index (HF), K: Infant mortality rate (InfMort), L: PA Size (GISAREAL), M: Corruption Perception Index (Corruption), N: Environmental Performance Index (EPI), O: Gini Coefficient (Income inequality), P: Human Development Index (HDI), Q: GDP PPP per capita 2008 (GDP)

Supplementary Tables

Supplementary Table 1. Sample size summary

The number of time series in each **(a)** taxonomic class and geographic region and **(b)** IUCN protected area category.

a

**Number of time series**	**Global**	**Birds**	**Mammals**
Africa	679	131	550
Asia	92	35	77
Australasia	62	87	12
Europe	694	758	102
Latin America and the Caribbean	28	2	25
North America	122	55	68
SUM	1902	1068	834

b

**IUCN Category**	**Sample Size (n)**
Ia	130
Ib	24
II	704
III	51
IV	532
V	294
VI	88
Unknown	79
SUM	1902

Supplementary Table 2. Summary of the hypotheses being tested by the explanatory variables considered in the models. The expected direction of the relationship between the variable and trends in population abundance is indicated as positive (+), neutral (=), negative (-) or unknown (?). Examples of previous work examining the variables are provided in the Examples column. Scale refers to resolution of the data used – (Pop) Individual populations, (PA) Protected Area, (SP) Species, (N) National.

Scale	Variable	Justification and Hypothesised Relationship	Expected Relationship	Examples
Pop	Data points in series	Test whether data quality biases trend, more highly sampled time series could be expected to originate from more intensively managed PAs	=	
Pop	Initial year of series	Test whether the starting year of a time series influences trends	= or +	[13]
Pop	Middle year of series (MidYear)	African time series are known to be more negative prior to 1990	+	[13]
Pop	Chronological length of time series	Test whether time series length influences trends	=	[13]
PA	PA Size (GISAREAL)	PA size has been linked to PA ability to retain species [14,15]	+	[16,17]
PA	Perimeter-Area ratio (PerArRatio)	PAs with greater edge have more boundary to define and enforce, also more prone to edge effects. [18,19]	-	[20]
PA	Shape Index (SHIDX)	Linked to area. Greater Shape Index (less like a circle) hypothesised to be inversely correlated with population trends. [19,21]	-	[22]
PA	PA Age (PAage)	Some PAs have been shown to improve outcomes through time. We hypothesize that older PAs will have more positive population trends [23]	+	[24]
PA	IUCN Category (IUCNCat; IUCNCatBin)	IUCN category reflects strictness of human land use restrictions and theoretically the level of protection provided [25]	-	[26]
SP	Taxonomic Class (Class)	Controls for the influence of class. Also we expected mammals were likely to have more negative outcomes, since there are a higher proportion of threatened mammals than birds [4,27]	Birds +	
SP	Body mass (BMass)	Larger, k-selected species at higher risk of extinction [28]	-	[3,29]
SP	Species Red List status (Threat; ThreatBin)	Threatened species will likely have more negative trends as reduced abundance is the most common reason for listing a species as threatened [27]	-	[27]
N	Human Development Index (HDI)	We hypothesise that more developed nations will have more positive population trends because more money will be available for PAs. Though in some nations increased PA isolation and encroachment associated with development may reduce wildlife abundance	+	[30]
N	Corruption Perception	Increased corruption may indicate weaker	+	[32]

	Index (Corruption)	enforcement of protected areas and reduced funding [31]		
N	Environmental Performance Index (EPI)	High levels of environmental awareness and management effectiveness are likely to correlate with better PA outcomes	+	[33]
N	GDP PPP per capita 2008 (GDP)	GDP increases both conservation capacity and development pressures [30]	?	[30]
N	Gini Coefficient (Income inequality)	Greater income inequality has been shown to be spatially correlated with increased numbers of threated species [34]	-	[34]
PA	Road Length (gROADS)	Roads have been shown to damage biodiversity both directly and through enabling access for further land use conversion [35]	-	[36]
PA	Human population density (GRUMP)	Local human population likely to negatively influence status of biodiversity [37]	-	[37]
PA	Human Influence Index (HII)	Local populations and built infrastructure likely to negatively influence status of biodiversity [37]	-	[38]
N	Infant mortality rate (InfMort)	Proxy for poverty, subnational scale. Countries with less poverty are likely to harvest resources from PAs less frequently [39]	-	[40]
PA	Travel time to urban areas (UrbanTravel)	Increasing remoteness from human centers likely to reduce threat intensity. [41-43]	+	[41,44]
PA	Agricultural Suitability (AgSuit)	More agriculturally suitable land is more likely to be converted, and PAs will be influenced by a cocktail of local stressors [45].	-	[45]
PA	Elevation (SRTM90MEANELEV)	Higher elevations often less agriculturally suitable and more remote reducing threat intensity[46]	+	[46,47]
PA	Slope (SRTM90MEANSLOPE)	Steeper slopes often less agriculturally suitable and more remote reducing threat intensity[46]	+	[44,46,47]
PA	Vector Ruggedness Measure (VRM)	More rugged landscapes are known to be more remote and less agricultural suitable, reducing threat intensity[46]	+	[46,47]
PA	Percentage Natural Land-cover in Buffer (Nat_%_LC)	More natural landcover in buffer region should increase the effective size of the PA and the population sizes of the species present. More important for some taxa. [48]	+	[49]
PA	Isolation Index	More isolated compared to surrounding area will correlate with worse outcomes [50]	-	[21]

Supplementary Table 3. Description of explanatory variables considered in the models.

Variable names used the main text and results tables are indicated in brackets. Variables are organised into six groups: (D) PA design (e.g. size, shape, IUCN management category), (SEC) socio-economic context of the region and country in which the PA is located (e.g. wealth, corruption), (SP) species' traits which might determine response (e.g. body mass), (LH) local human impacts (e.g. road-density, land-use change), (BP) biophysical context (e.g. PA Elevation), and (T) time-series characteristics (e.g. length) The Scale of the variable: (Pop) population, (Sp) species, (PA) individual protected area, (N) national. Type indicates the nature of the variable: continuous (cont), categorical (cat) or binary (bin).

Group	Scale	Variable	Type	Description	Sources
-		Dependent variable	Cont	Slope of the population times series, calculated as described in methods	Calculated from raw population estimates, see methods
T	Pop	Data points in series	Cont	Number of years with a population abundance measure in a series	Calculated from time series data.
T	Pop	Initial year of series	Cont	The first year with a population measure	Calculated from time series data.
T	Pop	Middle year of series (MidYear)	Cont	The middle year of a time series. This variable was standardised by subtracting the median value (1989) of MidYear from all values. Units: Years	Calculated from time series data.
T	Pop	Chronological length of time series	Cont	The number of years between the initial and final years in the time series. Units: years	Calculated from time series data.
D	PA	PA Size (GISAREAL)	Cont	Total area of each PA in km^2 using R and the geosphere *(41)* and sp *(45)* packages.	Calculated using data from the WDPA World Database on Protected Areas: 2012 Annual Release *(46)*
D	PA	Perimeter-Area ratio (PerArRatio)	Cont	PA perimeter in km divided by PA area in km^2 using R and the geosphere *(41)* and sp *(45)* packages.	Calculated using spatial data from the WDPA World Database on Protected Areas: 2012 Annual Release *(46)*.
D	PA	Shape Index (SHIDX)	Cont	Shape index calculated using the following formula: √(area/(π*(2*perimeter/π)^2)) in R, using the R geosphere *(41)* and sp *(45)* packages.	Calculated using spatial data from the WDPA World Database on Protected Areas: 2012 Annual Release *(46)*.
D	PA	PA Age (PAage)	Cont	Years since the establishment of the PA (first protection) subtracted from the year 2010. Units: years*	*Where available, this was obtained from the World Database on Protected Areas: 2012 Annual Release *(46)*. Where not recorded in the WDPA, this was obtained from managing agencies (including online agency repositories and reports, and offline
D	PA	IUCN Category	Cat	IUCN Category (I-VI)*	

		(IUCNCat)			agency databases). Where data was still unavailable, site managers were directly contacted.
D	PA	IUCN Category (IUCNCatBin)	Bin	IUCN Categories collapsed into two groups: I-IV and V+VI*	
SP	Sp	Taxonomic Class (Class)	Cat	Taxonomic Class: Aves, Mammalia	Following taxonomic classification in the IUCN Red List: IUCN 2014. *The IUCN Red List of Threatened Species. Version 2014.3*. <http://www.iucnredlist.org>. Downloaded on 17 November 2014.
SP	Sp	Body mass (BM)	Cont	Adult female body mass in grams where available, otherwise mean adult body mass	Mammals: PanTHERIA database, Jones et al. (2009), Ecology 90:2648<br>Birds: BirdLife International (2014) IUCN Red List for birds. Downloaded from http://www.birdlife.org on 17 November 2014<br><br>Missing values were sourced from AnAge, a global database of species-specific metadata where possible (http://genomics.senescence.info/species/), and otherwise a range of independent species-specific data sources.
SP	Sp	Species Red List status (Threat)	Cat	Red List status in 2008.	IUCN Red List: IUCN 2014. *The IUCN Red List of Threatened Species. Version 2014.3*. <http://www.iucnredlist.org>. Full range of categories (Categorical), and a reclassification (Binary) into Threatened (including Data Deficient) and Lower Risk species following the Red List protocol.
SP	Sp	Species Red List status (ThreatBin)	Bin	Red List status in 2008, collapsed into two groups: threatened (CR, EN, VU, DD) and not threatened (LC, NT)	
SEC	N	Human Development Index (HDI)	Cont	The value of the country's Human Development Index in 2005	United Nations Development Program: http://hdr.undp.org/en/statistics/
SEC	N	Corruption Perceptions Index (Corruption)	Cont	The value of the country's Corruption Perceptions Index in 2008	Transparency International: http://www.transparency.org/policy_research/surveys_indices/cpi/2008
SEC	N	Environmental Performance Index (EPI)	Cont	The value of the country's Environmental Performance Index in 2008	Yale University http://epi.yale.edu/
SEC	N	GDP PPP per capita 2008 (GDP)	Cont	Per capita Gross Domestic Product adjusted for Purchasing Power Parity. Units: 2008 US$	International Monetary Fund: http://www.imf.org/external/data.htm#data
SEC	N	Gini Coefficient (Income inequality)	Cont	Gini Market in 1995. A Gini coefficient of 0 represents perfect equality, while an index of 100	World Bank: http://data.worldbank.org/indicator/SI.POV.GINI

				implies perfect inequality.	
LH	PA	Road Length (gROADS)	Cont	Sums of the length of roads in the 5, 10 and 25 km buffers around the PA divided by the non-marine surface area of the respective buffers. The protected area polygon was first intersected with the gROADS layer in ArcGIS and the total road density was calculated in R using the geosphere *(41)* and sp packages (46).	Center for International Earth Science Information Network - CIESIN - Columbia University & Information Technology Outreach Services - ITOS - University of Georgia. Global Roads Open Access Data Set, Version 1 (gROADSv1). (2013). at <http://dx.doi.org/10.7927/H4VD6WCT>
LH	PA	Human population density (GRUMP)	Cont	Mean and median of the cell values for human population density in 1995, calculated in 5, 10, and 25km buffers around each PA (terrestrial area only) using R and the raster package (41). Minimum 3 raster cells overlapped	Center for International Earth Science Information Network - CIESIN - Columbia University, International Food Policy Research Institute - IFPRI, The World Bank & Centro Internacional de Agricultura Tropical - CIAT. Global Rural-Urban Mapping Project, Version 1 (GRUMPv1): Population Density Grid. (2011). at <http://dx.doi.org/10.7927/H4R20Z93><br><br>Balk, D. L. et al. Determining Global Population Distribution: Methods, Applications and Data. Adv. Parasitol. 62, 119–156 (2006).
LH	PA	Human Influence Index (HII)	Cont	Mean and median index value of all cells within buffers of 5, 10, and 25km surrounding the PA calculated using R and the raster package (41). Minimum 3 cells overlapped	Wildlife Conservation Society - WCS & Center for International Earth Science Information Network - CIESIN - Columbia University. Last of the Wild Project, Version 2, 2005 (LWP-2): Global Human Influence Index (HII) Dataset (IGHP). (2005). at <http://dx.doi.org/10.7927/H46W980H>
LH	N	Infant mortality rate (InfMort)	Cont	Mean value calculated in 5, 10, and 25km buffers (terrestrial area only) around each PA using R and the geosphere (41) and sp packages (45) to calculate the area weighted mean for the subnational-level data polygons for infant mortality rate (data from 1990-2002). Units: number of deaths before 1yr for every 1000 births.	Center for International Earth Science Information Network - CIESIN - Columbia University. Poverty Mapping Project: Global Subnational Infant Mortality Rates. (2005). at <http://dx.doi.org/10.7927/H4PZ56R2><br><br>Storeygard, A., Balk, D., Levy, M. & Deane, G. The Global Distribution of Infant Mortality: A subnational spatial view. Popul. Space Place 14, 209–229 (2008).
LH	PA	Travel time to urban areas (UrbanTravel)	Cont	Mean and median of cell values within the protected area polygon, representing travel time from that cell to the nearest urban settlement (> 50,000 people in	Nelson, A. Estimated travel time to the nearest city of 50,000 or more people in year 2000. Global Environment Monitoring Unit - Joint Research Centre of the European Commission, Ispra Italy.

				2000), calculated using R and the raster package (41). Minimum 1 cell overlapped.	(2008). at <http://forobs.jrc.ec.europa.eu/products/gam/index.php>
BP	PA	Agricultural Suitability (AgSuit)	Cont	Mean of cell values within the protected area polygon calculated using R and the raster package (41) for each data set. Minimum 1 cell overlapped.	Two data sets of agricultural suitability we used and analyzed independently:<br>1. Climate, soil and terrain slope constrains combined (Plate 28) in Fischer, G., van Velthuizen, H., Nachtergaele, F. & Medow, S. Global Agro-Ecological Zones. FAO/IIASA. (2000). at <http://webarchive.iiasa.ac.at/Research/LUC/GAEZ/index.htm><br>2. Combined suitability of global land area for pasture and rainfed crops (intermediate input level) (FGGD, Map 6.63) in van Velthuizen, H. et al. Mapping biophysical factors that influence agricultural production and rural vulnerability. FAO/IASA. Environ. Nat. Resour. Ser. 11, 1–93 (2007). at http://www.fao.org/docrep/010/a1075e/a1075e00.htm
BP	PA	Elevation (SRTM90MEANELEV)	Cont	Mean and median cell values within the protected area polygon (terrestrial area only) calculated using R and the raster *(40)*, rgdal *(44)* and rgeos *(43)* packages. Minimum 4 raster cells overlapped¨	*Jarvis, A., Reuter, H., Nelson, A. & Guevara, E. Hole-filled seamless SRTM data v4. International Centre for Tropical. Agriculture (CIAT). (2008). at <http://srtm.csi.cgiar.org>;<br>Polar areas (not covered by SRTM, above): Median elevation, 7.5 arc-sec grid (md_75) in Danielson, J. J. & Gesch, D. B. Global Multi-resolution Terrain Elevation Data 2010 (GMTED2010). U.S. Geological Survey Open-File Report 2011-1073. 26pp (2011). at <http://topotools.cr.usgs.gov/gmted_viewer/>.
BP	PA	Slope (SRTM90MEANSLOPE)	Cont	Mean and median of all slope values, calculated for each cell of the elevation raster, within the protected area polygon (terrestrial area only) using R and the raster *(40)*, rgdal *(44)* and rgeos *(43)* packages. Minimum 4 raster cells overlapped¨	
BP	PA	Vector Ruggedness Measure (VRM)	Cont	Mean and median cell values within the protected area polygon (terrestrial area only) for VRM cell values calculated using R and the raster *(40)*, rgdal *(44)* and rgeos *(43)* packages, with calculations based on (72).	
LH	PA	Percentage Natural Land-cover (Nat_%_LC)	Cont	The land-cover raster layers were first reclassified as in e.g. [51]: for each land-cover product using the R raster package (41), cells with obvious human	*Two different land-cover raster layers were used:<br>1. Defourny, P. et al. GlobCover: A 300M Global Land Cover Product for 2005 Using ENVISAT MERIS Time Series. in Proc. ISPRS

				influenced land-cover classes were considered ‘human-modified'; all others were ‘natural’. Reclassifications are provided in supplementary information table 4. The fraction of all cells within the protected area and those within buffers of 5, 10, and 25km surrounding the PA classified as 'natural' were calculated using R and the raster package (41) with each reclassified land-cover raster layer. Minimum 4 cells overlapped.	Comm. VII Mid-Term Symp. 8–11 (2007)<br><br>2.Land Processes Distributed Active Archive Center (LP DAAC), MCD12Q1 2005<br>MODIS Land Cover product dataset: NASA EOSDIS Land Processes DAAC, USGS<br>Earth Resources Observation and Science (EROS) Center, Sioux Falls.<br>South Dakota. (2005). at <https://lpdaac.usgs.gov/products/modis_products_table/mcd12q1>
BP	PA	Isolation Index	Cont	General measure of the difference between the land-cover class distribution in the PA and the corresponding 5, 10 and 25km buffer regions. R and the raster package (41) were used to calculate the general dissimilarity index (Czekanowski Index) between the land-cover class distributions of the PA and respective buffers for each land cover layer.*	

Supplementary Table 4. Rules used for reclassification and reweighting of land cover datasets in Supplementary Table 3

**GlobCover 300**				
**Raster Code**	**Reclassification**	**Weight**	**HFI Weight**	**Land Cover Description**
11	Human Modified	4	8	Post-flooding or irrigated croplands (or aquatic)
14	Human Modified	3	3	Rainfed croplands
20	Human Modified	2	2	Mosaic cropland (50-70%) / vegetation (grassland/shrubland/forest) (20-50%)
30	Human Modified	1	1	Mosaic vegetation (grassland/shrubland/forest) (50-70%) / cropland (20-50%)
40	Natural	0	0	Closed to open (>15%) broadleaved evergreen or semi-deciduous forest (>5m)
50	Natural	0	0	Closed (>40%) broadleaved deciduous forest (>5m)
60	Natural	0	0	Open (15-40%) broadleaved deciduous forest/woodland (>5m)
70	Natural	0	0	Closed (>40%) needleleaved evergreen forest (>5m)
90	Natural	0	0	Open (15-40%) needleleaved deciduous or evergreen forest (>5m)
100	Natural	0	0	Closed to open (>15%) mixed broadleaved and needleleaved forest (>5m)
110	Natural	0	0	Mosaic forest or shrubland (50-70%) / grassland (20-50%)
120	Natural	0	0	Mosaic grassland (50-70%) / forest or shrubland (20-50%)
130	Natural	0	0	Closed to open (>15%) (broadleaved or needleleaved, evergreen or deciduous) shrubland (<5m)
140	Natural	0	0	Closed to open (>15%) herbaceous vegetation (grassland, savannas or lichens/mosses)
150	Natural	0	0	Sparse (<15%) vegetation
160	Natural	0	0	Closed to open (>15%) broadleaved forest regularly flooded (semi-permanently or temporarily) - Fresh or brackish water
170	Natural	0	0	Closed (>40%) broadleaved forest or shrubland permanently flooded - Saline or brackish water
180	Natural	0	0	Closed to open (>15%) grassland or woody vegetation on regularly flooded or waterlogged soil - Fresh, brackish or saline water
190	Human Modified	4	10	Artificial surfaces and associated areas (Urban areas >50%)
200	Natural	0	0	Bare areas
210	NA	NA	NA	Water bodies
220	NA	NA	NA	Permanent snow and ice
230	NA	NA	NA	No data (burnt areas, clouds,…)
**MODIS Terra+Aqua Land Cover (MCD12Q1)**				
**Raster Code**	**Reclassification**	**Weight**	**HFI Weight**	**Land Cover Description**
0	NA	NA	NA	Water
1	Natural	0	0	Evergreen Needleleaf forest
2	Natural	0	0	Evergreen Broadleaf forest
3	Natural	0	0	Deciduous Needleleaf forest
4	Natural	0	0	Deciduous Broadleaf forest

5	Natural	0	0	Mixed forest
6	Natural	0	0	Closed shrublands
7	Natural	0	0	Open shrublands
8	Natural	0	0	Woody savannas
9	Natural	0	0	Savannas
10	Natural	0	0	Grasslands
11	Natural	0	0	Permanent wetlands
12	Human Modified	3	8	Croplands
13	Human Modified	4	10	Urban and built-up
14	Human Modified	2	3	Cropland/Natural vegetation mosaic
15	NA	NA	NA	Snow and ice
16	Natural	0	0	Barren or sparsely vegetated
254	NA	NA	NA	Unclassified
255	NA	NA	NA	Fill Value

Supplementary Table 5. Summary of model results

Table showing the significant fixed effect variables fitted in the preferred models for all datasets. The sign of the parameter estimate indicated by '+' or '-' with the significance levels, derived from highest posterior credibility intervals from MCMC methods, indicated by the number of '+' or '-': † p ≤ 0.1; + P ≤ 0.05; ++ P ≤ 0.01; +++ P ≤ 0.001. Each column gives the results from a separate dataset with the number of time series (n_{ts}) in that dataset followed by the number of species (n_{spp}) for which time series are available (n_{ts}, n_{spp}) in parentheses below the name of the subset. Sets of collinear variables that could be substituted for those in the most parsimonious model are indicated by letters: capital letters indicate the preferred variable, and lowercase letters indicate substitutable significant variables. Variables tested but found to show no substantial explanatory power in that dataset are indicated by 'ns'. Variables not tested in a dataset are indicated by 'na', this was either because they were inapplicable or missing values prevented testing. The level in the nested hierarchy of each variable is indicated by letter/s in parentheses in description of variables: N is National level metric, S is species level, and PA is PA-level.

**Variable**	**Global (1902, 556)**	**Description of variable**	**Mammals (834, 208)**	**Birds (1068, 348)**	**Africa (681,144)**	**Europe (694, 269)**
Class (Mammal)	- -	Taxonomic Class (S)	na	na	ns	-
Body Mass	+ + +	Species Body Mass (S)	+ + +	++	+	+ + +
BM^2	ns	Species Body Mass Squared (S)	+	ns	+ + +	ns
HDI	++ a	Human Development Index (N)	+ + + A	+ + A	ns	+ + A
HDI^2	ns	Human Development Index Squared (N)	ns	ns	ns	- -
HDI^3	ns	Human Development Index Cubed (N)	- -	ns	ns	ns
GDP	+ a	Gross Domestic Product (N)	+ + a	ns	ns	ns
EPI	+ + a	Environmental Performance Index (N)	+ + a	†a	ns	+ + a
Corruption	+ a	Corruption Index (N)	+ a	ns	ns	† a
Gini index	+ +	Income Inequality (N)	ns	++	ns	ns
Groads	ns	Road Network Length in 25km buffer (PA)	+ + B	ns	+ + +	ns
UrbanTravel	ns	Urban Travel Time (PA)	- b	ns	ns	ns
NatLC% (MODIS)	ns	Percentage of Natural Land Cover based on MODIS (PA)	- b	ns	ns	ns
HII in 25k (MODIS)	ns	Human Impact Index in 25km buffer based on MODIS (PA)	ns	ns	ns	† b
NatLC% (GLOBCOVER)	na	Percentage of Natural Land Cover in 25km buffer based on GLOBCOVER (PA)	na	na	na	+ + b
HII in 25k (GLOBCOVER)	na	Human Impact Index in 25km buffer based on GLOBCOVER (PA)	na	na	na	+ + B
MidYear	ns	The Central Year of the Time Series	ns	ns	+ + +	ns
MidYear^2	ns	The Central Year of the Time Series ^2	ns	ns	+ +	ns

Supplementary Table 6. Model parameter estimates

Tables showing parameter estimates and summary output for the most parsimonious suite of models (ΔAIC <4 compared to the best model) in each dataset: (a) Global, (b) Mammals subset, (c) Birds subset, (d) African subset and (e) Europe subset. 2nd and 3rd order polynomials are indicated by carats (^). All models include species, PA and country as random effects.

a. Global

Model ID	Intercep	BMass	BMass^	Class	Gini	HDI	AICc	ΔAIC	weight
30	0.0219	1.121	NA	+	0.596	0.9543	-2618.63	0	0.480
32	0.0235	1.183	0.1884	+	0.578	0.9808	-2618.19	0.436	0.386

b. Mammal

Model ID	Intercept	BMass	BMass^2	HDI	HDI^2	HDI^3	Groads	AICc	ΔAIC	weight
44	-0.0080	0.646	0.269	0.642	0.053	-0.469	0.447	-1144.46	0	0.442
62	-0.0089	0.650	NA	0.679	0.080	-0.481	0.428	-1143.26	1.20	0.242
40	-0.0043	0.636	0.282	0.588	NA	NA	0.436	-1141.21	3.25	0.086

c. Birds

Model ID	Intercept	BMass	Gini	HDI	AICc	ΔAIC	weight
8	0.01442	0.3986	0.4298	0.4931	-1474.9	0	0.7611

d. Africa

Model ID	Intercept	BMass	BMass^2	Class	Groads	Midyear	Midyear^2	AICc	ΔAIC	weight
64	0.1127	0.384	0.507	+	0.717	0.5986	0.384	-843.83	0	0.8147
48	0.0087	0.425	0.552	+	0.735	0.5857	NA	-840.5	3.30	0.1568

e. Europe

Model ID	Intercept	BMass	Class	HDI	HDI^2	MHIIL251	AICc	ΔAIC	weight
32	0.0229	0.7393	+	0.624	-0.432	0.315	-1274.97	0	0.487
16	0.0224	0.7298	+	0.519	-0.499	NA	-1273.67	1.294	0.255
24	0.0211	0.7426	+	0.671	NA	0.375	-1272.99	1.981	0.181

Supplementary Notes

When initially submitted the manuscript included models that used rates of Child Malnutrition as a predictor variable. During the review process it was requested that this variable be removed because the data were at sub-national spatial scale unlike the remaining socioeconomic predictor variables. The authors complied with this request. Removal of this predictor variable did not meaningfully change the results because the data on rates of Child Malnutrition were highly correlated with national Human Development Index scores. In the interests of transparency, the parameter estimates of the preferred Global models are presented below, both with, and without, Child Malnutrition as a predictor variable. It should be noted that the sample size and other parameter estimates remain stable in both cases.

Preferred Global model with Child Malnutrition Fitted

Model ID	Intercept	BMass	BMass^2	Class	Gini	Malnutrition % in 25km	AICc	ΔAIC	weight
22	0.0195	1.090	NA	+	0.63	-1.064	-2620.1	0	0.5095
32	0.0211	1.153	0.1924	+	0.61	-1.093	-2619.8	0.36	0.4237

Preferred Global model without Malnutrition fitted (same as in SI above)

Model ID	Intercep	BMass	BMass^2	Class	Gini	HDI	AICc	ΔAIC	weigh
30	0.0219	1.121	NA	+	0.596	0.9543	-2618.63	0	0.480
32	0.0235	1.183	0.1884	+	0.578	0.9808	-2618.19	0.436	0.386

Supplementary References

1 Blackburn, T. I. M. & Gaston, K. The distribution of mammal body masses. *Divers. Distrib.* **4**, 121-133, doi:10.1046/j.1365-2699.1998.00015.x (1998).

2 Dunning, J. B. J. *The CRC Handbook of Avian Body Masses, 2nd edition*. (2008).

3 Cardillo, M. *et al.* The predictability of extinction: biological and external correlates of decline in mammals. *Proc. R. Soc. Biol. Sci. Ser. B* **275**, 1441-1448 (2008).

4 Cardillo, M. *et al.* Multiple Causes of High Extinction Risk in Large Mammal Species. *Science* **309**, 1239-1241, doi:10.1126/science.1116030 (2005).

5 Davidson, A. D., Hamilton, M. J., Boyer, A. G., Brown, J. H. & Ceballos, G. Multiple ecological pathways to extinction in mammals. *Proc. Natl. Acad. Sci. U. S. A.* **106**, 10702-10705 (2009).

6 Isaac, N. J. B. & Cowlishaw, G. How species respond to multiple extinction threats. *Proc. R. Soc. Biol. Sci. Ser. B* **271**, 1471-2954 (2004).

7 Stoner, C. *et al.* Changes in large herbivore populations across large areas of Tanzania. *Afr. J. Ecol.* **45**, 202-215, doi:doi:10.1111/j.1365-2028.2006.00705.x (2007).

8 Owens, I. P. F. & Bennett, P. M. Ecological basis of extinction risk in birds: Habitat loss versus human persecution and introduced predators. *Proc. Natl. Acad. Sci. U. S. A.* **97**, 12144-12148 (2000).

9 Pocock, M. J. O. Can traits predict species' vulnerability? A test with farmland passerines in two continents. *Proc. R. Soc. Biol. Sci. Ser. B* **278**, 1532-1538, doi:10.1098/rspb.2010.1971 (2011).

10 Fritz, S. A., Bininda-Emonds, O. R. P. & Purvis, A. Geographical variation in predictors of mammalian extinction risk: big is bad, but only in the tropics. *Ecol. Lett.* **12**, 538-549 (2009).

11 Grier, J. Ban of DDT and subsequent recovery of Reproduction in bald eagles. *Science* **218**, 1232-1235, doi:10.1126/science.7146905 (1982).

12 Eaton, M. A. *et al.* The state of the UK's birds 2012. (RSPB, BTO, WWT, CCW, NE, NIEA, SNH and JNCC, Sandy, Bedfordshire, 2012).

13 Craigie, I. D. *et al.* Large mammal population declines in Africa's protected areas. *Biol. Conserv.* **143**, 2221-2228 (2010).

14 MacArthur, R. H. & Wilson, E. O. *The Theory of Island Biogeography*. (Princeton, 1967).

15 Newmark, W. D. A land-bridge island perspective on mammalian extinctions in western North American Parks. *Nature* **325**, 430-432 (1987).

16 Newmark, W. D. Extinction of mammal populations in western North American national parks. *Conserv. Biol.* **9**, 512-526 (1995).

17 Laurance, W. F. *et al.* Averting biodiversity collapse in tropical forest protected areas. *Nature* **489**, 290-294 (2012).

18 Leverington, F. *et al.* Management effectiveness evaluation in protectedareas – a global study. Second edition. (The University of Queensland Brisbane AUSTRALIA, 2010).

19 Kunin, W. E. Sample shape, spatial scale and species counts: Implications for reserve design. *Biol. Conserv.* **82**, 369-377 (1997).

20 Nelson, A. & Chomitz, K. M. Effectiveness of Strict vs. Multiple Use Protected Areas in Reducing Tropical Forest Fires: A Global Analysis Using Matching Methods. *PLoS ONE* **6**, e22722 (2011).

21 Ewers, R. M. & Didham, R. K. The effect of fragment shape and species sensitivity to habitat edges on animal population size. *Conserv. Biol.* **21**, 926-936 (2007).

22 Joppa, L. N. & Pfaff, A. Global protected area impacts. *Proc. R. Soc. Biol. Sci. Ser. B* **278**, 1633-1638, doi:10.1098/rspb.2010.1713 (2011).

23 Edgar, G. J., Barrett, N. S. & Stuart-Smith, R. D. Exploited reefs protected from fishing transform over decades into conservation features otherwise absent from seascapes. *Ecol. Appl.* **19**, 1967-1974 (2009).

24 Geldmann, J. *et al.* Changes in protected area management effectiveness over time: A global analysis. *Biol. Conserv.* **191**, 692-699, doi:10.1016/j.biocon.2015.08.029 (2015).

25 Dudley, N. *Guidelines for Applying Protected Area Management Categories*. (IUCN, 2008).

26 Ferraro, P. J. *et al.* More strictly protected areas are not necessarily more protective: evidence from Bolivia, Costa Rica, Indonesia, and Thailand. *Environmental Research Letters* **8**, 025011 (2013).

27 IUCN. *The IUCN Red List of Threatened Species version 2014.3*, <http://www.iucnredlist.org> (2014).

28 Cardillo, M. Biological determinants of extinction risk: why are smaller species less vulnerable? *Anim. Conserv.* **6**, 63-69 (2003).

29 Fritz, S. A. & Purvis, A. Phylogenetic diversity does not capture body size variation at risk in the world's mammals. *Proc. R. Soc. Biol. Sci. Ser. B* (2010).

30 Waldron, A. *et al.* Targeting global conservation funding to limit immediate biodiversity declines. *Proc. Natl. Acad. Sci. U. S. A.* **110**, 12144-12148, doi:10.1073/pnas.1221370110 (2013).

31 Smith, R. J., Biggs, D., St. John, F. A. V., ‘t Sas-Rolfes, M. & Barrington, R. Elephant conservation and corruption beyond the ivory trade. *Conserv. Biol.* **29**, 953-956, doi:10.1111/cobi.12488 (2015).

32 Smith, R. J., Muir, R. D. J., Walpole, M. J., Balmford, A. & Leader-Williams, N. Governance and the loss of biodiversity. *Nature* **426**, 67 - 70 (2003).

33 Ceddia, M. G., Bardsley, N. O., Gomez-y-Paloma, S. & Sedlacek, S. Governance, agricultural intensification, and land sparing in tropical South America. *Proc. Natl. Acad. Sci. U. S. A.* **111**, 7242-7247, doi:10.1073/pnas.1317967111 (2014).

34 Mikkelson, G. M., Gonzalez, A. & Peterson, G. D. Economic Inequality Predicts Biodiversity Loss. *PLoS ONE* **2**, e444, doi:10.1371/journal.pone.0000444 (2007).

35 Laurance, W. F. *et al.* A global strategy for road building. *Nature* **513**, 229-232, doi:10.1038/nature13717 (2014).

36 Clements, G. R. *et al.* Where and How Are Roads Endangering Mammals in Southeast Asia's Forests? *PLoS ONE* **9**, e115376, doi:10.1371/journal.pone.0115376 (2014).

37 Luck, G. W. A review of the relationships between human population density and biodiversity. *Biological Reviews* **82**, 607-645 (2007).

38 Barber, C. P., Cochrane, M. A., Souza Jr, C. M. & Laurance, W. F. Roads, deforestation, and the mitigating effect of protected areas in the Amazon. *Biol. Conserv.* **177**, 203-209, doi:http://dx.doi.org/10.1016/j.biocon.2014.07.004 (2014).

39 Adams, W. M. *et al.* Biodiversity conservation and the eradication of poverty. *Science* **306**, 1146-1149 (2004).

40 Adams, W. M. & Hutton, J. People, parks and poverty: political ecology and biodiversity conservation. *Conservation and society* **5**, 147 (2007).

41 Ahrends, A. *et al.* Predictable waves of sequential forest degradation and biodiversity loss spreading from an African city. *Proc. Natl. Acad. Sci. U. S. A.*, doi:10.1073/pnas.0914471107 (2010).

42 Sanderson, E. W. *et al.* The human footprint and the last of the wild. *Bioscience* **52**, 891-904 (2002).

43 Lindsey, P. A. *et al.* The bushmeat trade in African savannas: Impacts, drivers, and possible solutions. *Biol. Conserv.* **160**, 80-96, doi:http://dx.doi.org/10.1016/j.biocon.2012.12.020 (2013).

44 Geldmann, J., Joppa, L. N. & Burgess, N. D. Mapping Change in Human Pressure Globally on Land and within Protected Areas. *Conserv. Biol.* **28**, 1604-1616, doi:10.1111/cobi.12332 (2014).

45 Henle, K. *et al.* Identifying and managing the conflicts between agriculture and biodiversity conservation in Europe–A review. *Agriculture, Ecosystems & Environment* **124**, 60-71, doi:http://dx.doi.org/10.1016/j.agee.2007.09.005 (2008).

46 Joppa, L. N. & Pfaff, A. High and far: biases in the location of protected areas. *PLoS ONE* **4**, e8273 (2009).

47 Margules, C. R. & Pressey, R. L. Systematic conservation planning. *Nature* **405**, 243 - 253 (2000).

48 Prugh, L. R., Hodges, K. E., Sinclair, A. R. E. & Brashares, J. S. Effect of habitat area and isolation on fragmented animal populations. *Proc. Natl. Acad. Sci. U. S. A.* **105**, 20770-20775 (2008).

49 Ferraz, G. *et al.* A large-scale deforestation experiment: effects of patch area and isolation on amazon birds. *Science* **315**, 238-242 (2007).

50 DeFries, R., Hansen, A., Turner, B. L., Reid, R. & Liu, J. LAND USE CHANGE AROUND PROTECTED AREAS: MANAGEMENT TO BALANCE HUMAN NEEDS AND ECOLOGICAL FUNCTION. *Ecol. Appl.* **17**, 1031-1038 (2007).

51 Joppa, L. N., Loarie, S. R. & Pimm, S. L. On the protection of "protected areas". *Proc. Natl. Acad. Sci. U. S. A.* **105**, 6673-6678, doi:10.1073/pnas.0802471105 (2008).
